# Comparison of Isogenic Strains Shows No Evidence of Altered Nosocomial Transmission-Competency of Rough, GPL-Negative Mycobacterium abscessus Strains

**DOI:** 10.1128/spectrum.01990-21

**Published:** 2022-03-21

**Authors:** Michal Meir, Mark Foreman, Michal Bar-Oz, Noga Naor, Anna Rozenblit, Daniel Barkan

**Affiliations:** a The Ruth Rappaport Children's Hospital, Rambam Medical Center, Haifa, Israel; b Koret School of Veterinary Medicine, The Robert H. Smith Faculty for Agriculture, The Hebrew University of Jerusalem, Rehovot, Israel; Emory University School of Medicine

**Keywords:** *Mycobacterium abscessus*, infection control, transmission, virulence

## Abstract

Mycobacterium abscessus is an emerging pathogen causing severe pulmonary infections. While environmental in origin, in the clinical setting M. abscessus often changes to a Rough phenotype associated with severe non-remitting infections. Clinical isolates baring mutations in glycopeptidolipid-synthesis genes, leading to the Rough phenotype, were suggested to have increase bacterial virulence while possibly showing reduced transmissibility on fomites. We set to determine whether an isolated glycopeptidolipid (GPL) defect affects transmissibility. We used transposon technology to create a fully isogenic Rough (GPL-defective) (Tn_4099c) and compare it to the isogenic parent strain (ATCC 19977). Survival on fomites was determined by spotting, drying, and retrieving the isolates at designated time points. This was repeated as a competition experiment using a mixture of differentially fluorescent M. abscessus *19977* (Smooth) and the Tn_4099c mutant (Rough). Survival ability in chlorhexidine solution (Septal Scrub Teva) was performed using a disinfectant killing-assay for mycobacteria. Despite significant bacterial killing in all assays, we found no survival advantage to either GPL-defected Rough or GPL-reserved Smooth morphotype—both on fomites and in chlorhexidine. Our findings suggest that while transmission fitness may be altered due to some within-host evolutionary changes, decreased transmissibility of clinical strains cannot be attributed to the GPL-synthesis defect alone. Further studies are needed to determine the effect of other mutations on the transmission potential of M. abscessus in the clinical setting.

**IMPORTANCE** Mycobacterium abscessus is an emerging pathogen causing severe pulmonary infections. In the clinical setting, M. abscsssus undergoes molecular and genetic changes associated with increased virulence. Specifically, bacterial defects in glycopeptidolipid (GPL) synthesis, creating the “Rough” colony phenotype, have been associated with increased virulence, yet were also presumably observed to have decreased survival on fomites, leading to reduced transmissibility. We set to determine whether GPL-synthesis defects are indeed responsible for reduced transmissibility of clinical isolates. We compared fully isogenic GPL-disrupted versus GPL-preserved strains, and demonstrated no survival advantage for either strain on fomites. Additionally, neither isolate had a survival advantage in chlorhexidine, a widely used disinfectant in health care settings. Our findings suggest that reduced transmissibility of clinical isolates, should it be found, cannot be attributed to GPL-synthesis mutations. While clinical isolates may show changes in transmission potential, more studies are needed to investigate the mechanisms leading to these phenotypic changes.

## INTRODUCTION

Mycobacterium abscessus is an emerging pathogen, causing severe pulmonary infections. Like most non-tuberculous mycobacteria, M. abscessus is considered an environmental organism with inherent abilities to survive harsh conditions and create biofilms. However, in clinical settings, M. abscessus often changes phenotypes further (1), with increasing virulence-associated features. One example to such phenotypic change is the transition from an initial smooth colony (S) morphotype to a rough colony morphotype (R), associated with increased virulence-associated phenotypes. This transition is almost uniformly related to loss of glyco-peptido-lipids (GPL) in the outer cell wall, due to abrogation of synthesis (inactivating mutations in the 20 kb GPL complex, *MAB_4098c* [*mps2*], *MAB_4099c* [*mps1*]) or GPL transport (*mmpL4b*) (2, 3). Other mechanisms of transition have been described, but are considerably rarer. Whether patients are first infected by environmental isolates, or by isolates transmitted from other infected individuals, is an unresolved question. Whole genome sequencing (WGS) analysis of a global collection of clinical isolates has suggested many acquisitions of M. abscessus infections are humanly transmitted through direct contact, aerosol, or fomites (4). However, other epidemiologic and WGS analyses have shown that while patient-to-patient transmission exists, it does not account for the majority of cases of M. abscessus infections among cystic fibrosis patients (5–8).The question of infectivity and spread of different isolates is therefore of paramount importance to infection-control measures in hospitals and outpatient clinics, especially in highly susceptible populations.

It was recently suggested that M. abscessus undergoes pathogenic evolutionary changes within infected hosts, increasing virulence features while diminishing transmission abilities (1). Specifically, rough colony patient-derived isolates with GPL mutations were shown to have considerably decreased survival on fomites compared to smooth controls, suggesting GPL mutations and rough phenotype decreases potential for nosocomial spread. Challenging this hypothesis, we used our verified GPL-defective transposon-mutant and isogenic parent-strain controls to examine whether indeed an isolated GPL defect affects transmissibility, and found no evidence to support this hypothesis.

## RESULTS

### Construction of the GPL-defected mutant.

**FIG 1:**
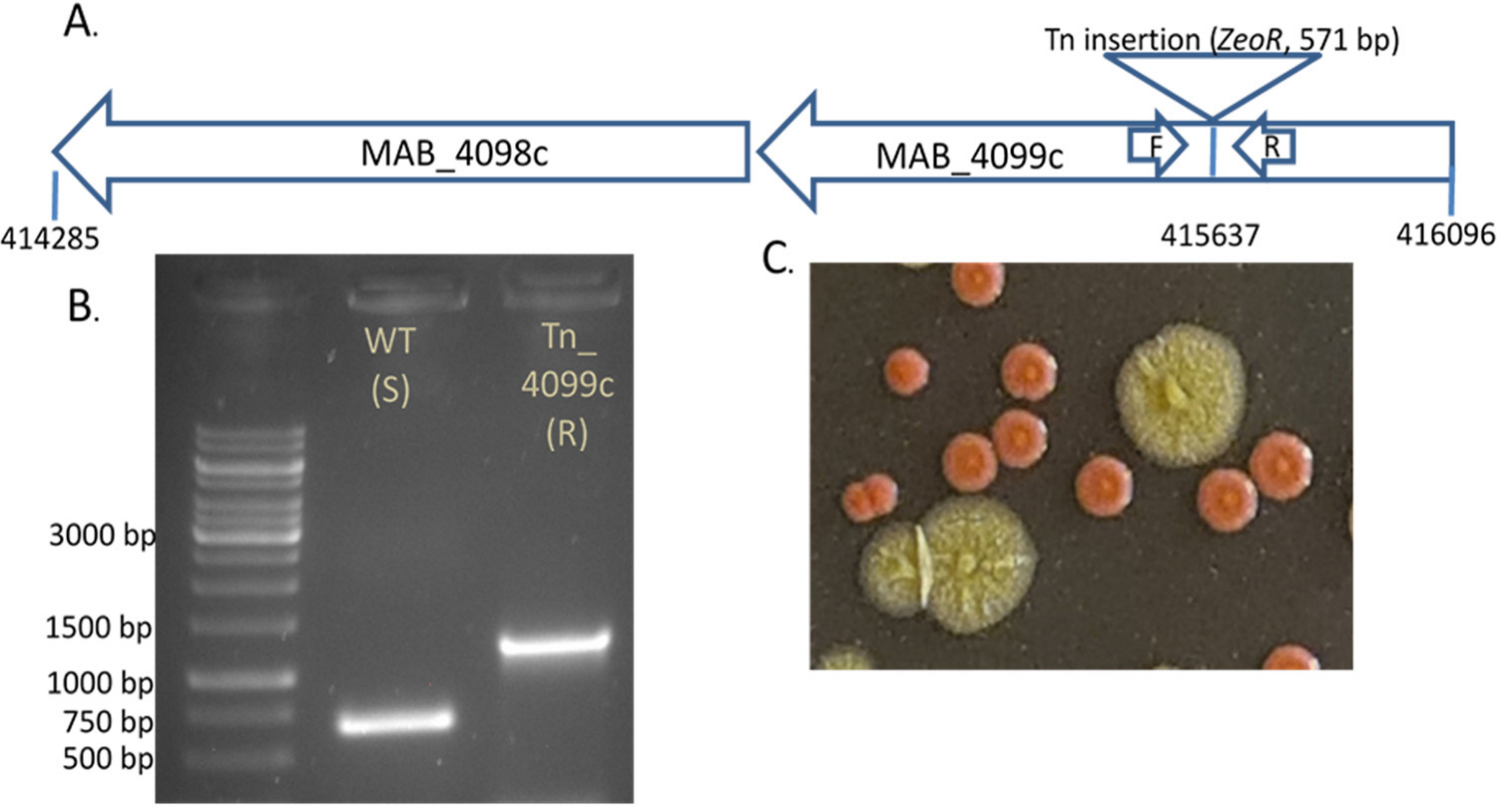
Construction of a MAB_4099c inactivated transposon mutant. (A) Schematic representation of the genomic organization for the GPL complex. The insertion point for the 571 bp transposon is shown. (B) PCR using primers F (5′ – TGGAACTCAATGCCGGCACCATGGA – 3′) and R (5′ – TGTGCGGTCAGGACGAGCCGATGCTT – 3′) (shown in A) was performed on the wild type (WT) and the Tn_4099c mutant. The expected product of ∼650 bp (in WT) is longer in the Tn-mutant by the length of the transposon, confirming the insertion point. For further confirmation, the 1,200 bp product was sequenced by Sanger sequencing. (C) For unequivocal identification, *ATCC19977* (S) was transfected with a red-fluorescence expressing plasmid (tdTomato), whereas the Tn_4099c mutant was transfected with an identical plasmid, expressing green fluorescence (mWasabi). Both fluorescent mutants are shown here, exemplifying how an unequivocal identification can be made.

We recently constructed and published a saturated transposon mutant library based on M. abscessus ATCC19977 of smooth colony morphotype (9). We isolated a rough colony Tn_mutant and verified it to be a GPL-complex defective mutant. The Tn insertion was mapped to base 4156378, inactivating the *MAB_4099c* (and the downstream *MAB_4098c*) genes (Fig. 1A), and its site was verified by PCR and Sanger sequencing of that locus (Fig. 1B). The parent strain ATCC19977 smooth wild type was used as isogenic control.

### Defects in GPL synthesis do not affect survival of M. abscessus on fomites.

**FIG 2:**
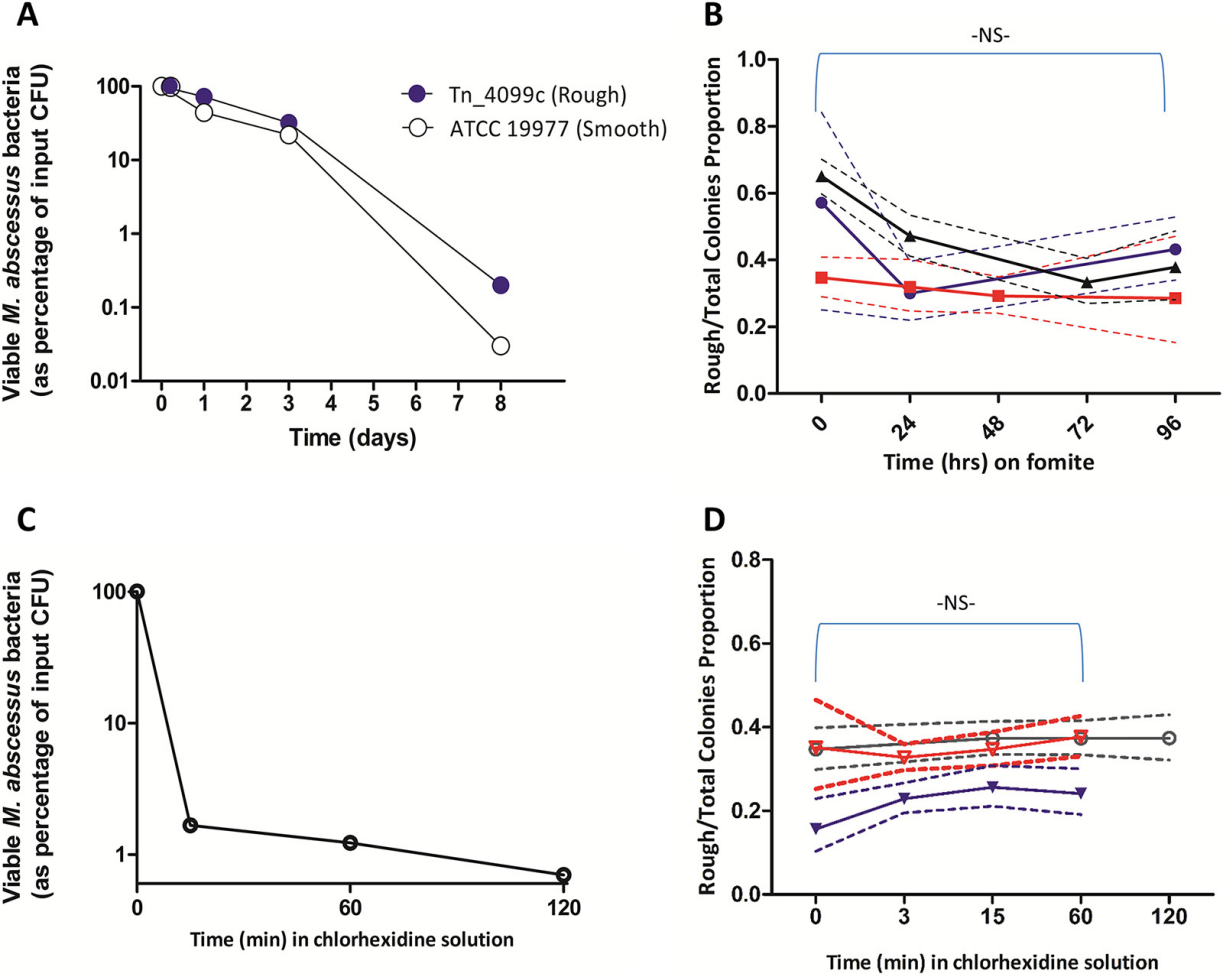
Survival dynamics of Smooth and Rough isolates on fomites and in chlorhexidine. (A) Survival of M. abscessus *ATCC 19977* and the Tn_4099c mutant after spotting on a sterile plastic 24-well plate was examined at designated time points. (B) A mixture of fluorescent M. abscessus *19977*^tdTomato^ (Smooth,) and the Tn_4099c^mWasabi^ mutant (Rough,), was spotted in a 24-well plate, and viable CFU number was determined as described above. The proportion of Red:Green bacteria was assessed at designated time points. Two hundred to 400 colonies were examined at each time point. The results of three independent experiments, each with a slightly different initial Red:Green ratio, are shown. The Wilson score 95% confidence interval is shown in dashed lines. The combined *P* value of all three experiments does not reach significance (> 0.05), and the 99% CI is 0.94–1.44. (C) Killing of M. abscessus by a commercial chlorhexidine solution diluted to a concentration of 1% is shown. One (representative) calibration experiment out of two is shown. (D) A mixture of fluorescent M. abscessus *19977*^tdTomato^ (Smooth) and the Tn_4099c^mWasabi^ mutant (Rough,) was placed in chlorhexidine 1%, and the ratio of S:R viable colonies was determined at designated time points, in three separate experiments. The Wilson score 95% confidence interval is shown in dashed lines; the combined *P* value for the three experiments is 0.12, with a 99% CI of 0.72–1.08.

Fomite survival experiments comparing rough GPL-defected to smooth isogenic isolates were performed similarly to experiments described by Bryant et al. (1). In contrast to these previous findings, we observed no significant difference in survival on fomites between the smooth wild type and the rough *Tn_4099c* mutant (Fig. 2A). To confirm our findings, we further conducted competition experiments, where both the R and S isolates were mixed, spotted together in several wells, and the S:R ratio was determined by plating on agar plates at designated time points (T = 0, 24h, 48h, etc.). To allow unequivocal identification of the S versus R isolates, we transfected them with a kanamycin-selected fluorescent plasmid (10) (the S variant with tdTomato-Red, and the R with mWassabi-Green, both a kind gift from Kevin Takaki and Lalita Ramakrishnan, Cambridge, United Kingdom) (Fig. 1C). The plasmids are identical, except the fluorescent protein itself. In repeated experiments, and regardless of the initial S:R ratio, neither isolate had a survival advantage, and the S:R ratio remained virtually unchanged until no viable colonies could be retrieved from the dry wells (Fig. 2B). Survival dynamics of the mixed bacteria on fomites (data not shown) were similar to those of separately spotted strains (Fig. 2A), demonstrating a substantial reduction in the total colony counts over time, thus excluding the possibility that the unchanged ratio was due to conditions favoring bacterial survival and minimal bacterial death.

### Defects in GPL synthesis do not affect survival of M. abscessus in chlorhexidine.

To evaluate the ability of rough isolates to survive a common disinfectant, we used Septal Scrub Teva, a commercial chlorhexidine-based soap. We first calibrated the system to find a chlorhexidine concentration producing effective, yet not complete killing of bacteria (99% killing) over 5-, 15-, and 60-min exposure at room temperature (24°C). This was found to be Septal Scrub diluted ×4 in water, to a concentration of 1% chlorhexidine-gluconate (Fig. 2C). We then conducted three independent competition experiments as the ones described for survival on fomites using an M. abscessus ATCC19977^tdTomato^ and a Tn_4099c^mWasabi^ mutant as above. The GPL-defected transposon mutant showed comparable survival to its isogenic counterpart (Fig. 2D).

## DISCUSSION

It has become evident that M. abscessus strains undergo genetic changes within infected hosts, thereby increasing virulence and drug resistance (11, 12). Transition from smooth to rough colony morphotypes, most commonly associated with genetic changes leading to a GPL-synthesis defect, is associated with increased virulence and nonremitting, debilitating disease (13). Recently, a genetic GPL-synthesis defect was reported to be associated with decreased survival of M. abscessus on fomites, suggesting GPL-defect mutations lead to reduced transmissibility (1). In contrast to these findings, our data show that defects in GPL synthesis are not necessarily associated with decreased survival on fomites, nor with increased susceptibility to chlorhexidine. Our findings complement a recent study, noting comparable aerosol transmission of smooth and rough isolates (14). Although it would seem conceivable that GPL content would influence properties of the cell wall in ways that would affect these parameters, our experimental results (and those in reference 14) conclusively show this is not the case. While differences among studies could be attributed to choice of specific isolates or seemingly small but potentially significant technical differences (like our use of PBS-Tween for resuspension of bacteria after drying, or declumping using a 27G needle before experiments), our findings challenge the generalization of the rule regarding reduced transmissibility of clinically-evolved isolates. Specifically, while transmission fitness may be altered due to some within-host evolutionary changes, decreased transmissibility of clinical strains cannot be attributed to the GPL-synthesis defect alone.

Our results continue to encourage common infection control practices, including appropriate drying and disinfection of respiratory equipment (inhalers and lung function respirators, etc.) to minimize the possibility of patient-to-patient transmission. Further studies are needed to conclude which mutations in clinical isolates, if any, truly contribute to altered transmission potential.

## MATERIALS AND METHODS

M. abscessus 19977, obtained from ATCC, was grown in Middlebrook 7H9 media, supplemented by 0.05% Tween80 and 0.05% glycerol. The Tn_*4099c* mutant was isolated from our previously generated Tn-mutant library (9). Declumping by passage through a 27G needle was performed before experiments to minimize plating and O.D600 errors. To determine survival on fomites, M. abscessus *ATCC 19977* and the Tn_4099c mutant were spotted (50 μL of an O.D_600_ culture, which translates to roughly 5 × 10^6^ CFU) in wells of a standard 24-well plastic plate and placed in a 37°C nonhumidified incubator. The wells dried in ∼2 h. At designated time points, bacteria were resuspended in 500 μL of saline/Tween80 (0.05%) for 10 min, pipetted repeatedly, serially diluted in saline/Tween80, and plated on Middlebrook 7H10 solid agar supplemented with 0.05% glycerol. Following 5–7 days, colonies were enumerated to calculate the number of viable CFU.

Competition experiments were performed using a mixture of fluorescent M. abscessus *19977* (Smooth, tdTomato fluorescence) and the Tn_4099c mutant (Rough, mWasabi fluorescence). Viable CFU numbers of each strain were determined as described above. The proportion of Red:Green bacteria was assessed at 24, 48, 72, and 96 h.

Survival ability in chlorhexidine solution was performed using a disinfectant killing-assay for mycobacteria (15). Septal Scrub (Teva), a commercial chlorhexidine-based (chorehexidine gluconate 4%, diluted in water) solution, was used. M. abscessus ATCC19977^tdTomato^ and a MAB Tn_4099c^mWasabi^ were mixed and placed into the disinfecting solution in a 1:10 volume ratio. The inoculated disinfectant solution was then neutralized with saline at 3, 15, 60, and 120 min, and plated in serial dilution to assess survival.

### Data availability.

All data are presented in the manuscript. Additional data, as well as materials, may be requested directly from the authors.
